# Synergistic effects of sequential infection with highly pathogenic porcine reproductive and respiratory syndrome virus and porcine circovirus type 2

**DOI:** 10.1186/1743-422X-10-265

**Published:** 2013-08-26

**Authors:** Peihu Fan, Yanwu Wei, Longjun Guo, Hongli Wu, Liping Huang, Jianbo Liu, Changming Liu

**Affiliations:** 1 Division of Swine Infectious Diseases, State Key Laboratory of Veterinary Biotechnology, Harbin Veterinary Research Institute, The Chinese Academy of Agricultural Sciences, 427 Maduan Street, Nangang District, Harbin 150001, China

**Keywords:** Pigs, Highly pathogenic porcine reproductive and respiratory syndrome virus, Porcine circovirus type 2, Sequential infection, Pathogenicity

## Abstract

**Background:**

Porcine reproductive and respiratory syndrome virus (PRRSV) is the causative agent of porcine reproductive and respiratory syndrome (PRRS) and porcine circovirus type 2 (PCV2) is associated with postweaning multisystemic wasting syndrome (PMWS) in pigs. Coinfection with highly pathogenic PRRSV (HP-PRRSV) and PCV2 in the field has recently become extensive in some Asian countries. A synergistic pathogenicity between PRRSV and PCV2 infections has previously been reported. However, the consequences of the sequential infection of pigs with these two viruses are unknown.

**Methods:**

Thirty 35-day-old piglets were randomly divided into six groups (n = 5 each): HP-PRRSV/PCV2 (group 1, inoculated with HP-PRRSV, then inoculated with PCV2 one week later), PCV2/HP-PRRSV (group 2, inoculated with PCV2, then inoculated with HP-PRRSV one week later), HP-PRRSV+PCV2 (group 3, inoculated with HP-PRRSV and PCV2 concurrently), HP-PRRSV (group 4, inoculated with HP-PRRSV), PCV2 (group 5, inoculated with PCV2), and the control (group 6, uninfected). This experiment lasted 28 days. Clinical symptoms and rectal temperatures were recorded each day after inoculation, body weight was recorded weekly, and serum samples were obtained for viral nucleic acid quantification and antibody titration. Variations in CD3^+^, CD4^+^ CD8^–^, CD3^+^, CD4^–^, and CD8^+^ cells, natural killer (NK) cells, and mononuclear cells were determined by flow cytometry. The serum concentrations of interferon γ (IFN-γ), tumor necrosis factor α (TNF-α), interleukin 10 (IL-10), and macrophage granulocyte-colony stimulating factor (GM-CSF) were determined. Pathological changes in different tissues from the experimentally infected pigs were recorded.

**Results:**

The piglets in group 1 had the highest viral loads, the lowest antibody titers, the most-severe clinical signs, and the highest mortality (3/5, 60%; the mortality in the other groups was 0%), and interstitial pneumonia was more severe in this group compare to the other HP-PRRSV infected groups. The serum levels of IFN-γ, TNF-α, IL-10, and GM-CSF varied (increased or decreased) most widely in group 1, as did each immunocyte subgroup.

**Conclusions:**

HP-PRRSV infection followed by PCV2 infection enhanced the replication of both viruses in the experimental piglets and led to more-severe clinical signs and lesions, indicating greater synergistic effects during the sequential infection of piglets with HP-PRRSV and then PCV2.

## Background

Porcine reproductive and respiratory syndrome virus (PRRSV) infections are characterized clinically by reproductive failure, including weak neonatal piglets, abortion, stillbirths, and mummified fetuses, impaired respiration, and high mortality [1]. PRRSV was first reported in the USA in 1987 [2], and first isolated in the Netherlands [3]. It was detected in China in 1996. A PRRSV mutant strain with a 90-nucleotide deletion in the gene encoding the viral nonstructural protein 2 has prevailed in South China since 2006. This strain, highly pathogenic (HP)-PRRSV, causes high fever and high morbidity and mortality in pigs and is responsible for severe economic losses in the pork industry [4-6]. Porcine circovirus type 2 (PCV2) causes postweaning multisystemic wasting syndrome (PMWS) [7], which is characterized by any or a combination of the following clinical signs: progressive wasting, anemia, lymphadenopathy, pneumonia, nephritis, and hepatitis in weaned piglets. PMWS was first described in western Canada in 1991 [8] and subsequently in other countries [9-12]. Mixed PRRSV and PCV2 infections have been reported [13,14] and have attracted widespread attention. Because coinfection with PRRSV and PCV2 is common in the pig populations of China, an in-depth understanding of the synergistic pathogenicity of the two viruses is vital. In 2000, Allan et al. [15] reported that PRRSV and PCV2 coinfection enhanced PCV2 replication, with no significant effect on PRRSV, and in 2001, Harms et al. [16] showed that PCV2 can increase the severity of the interstitial pneumonia caused by PRRSV during coinfection with the viruses. Rovira et al. [17] reported that pigs first infected with PRRSV and seven days later with PCV2 developed a more-severe clinical disease and more macroscopic and microscopic lesions. The objective of the present study was to determine the synergistic effects of sequential infection with HP-PRRSV and PCV2, in animals coinfected with HP-PRRSV and PCV2 in different sequences.

## Results

### Clinical signs

**Figure 1:**
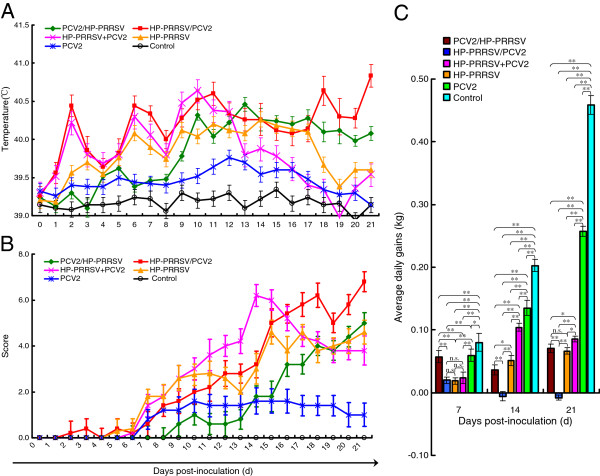
**Variation in mean rectal temperatures, scores for main clinical signs, and body weights in each infected group. (A)** The average rectal temperature of the HP-PRRSV/PCV2 group (18–21 dpi) was significantly higher than that of the PCV2/HP-PRRSV sequentially infected group, the HP-PRRSV+PCV2 group, or the HP-PRRSV group. The temperatures of the uninfected control group and the PCV2 group were normal. **(B)** Variations in the mean clinical sign scores. The mean score is the sum of five individual scores, each ranging from 0 to 2, resulting in a final score that ranges from 0 to 10 (0 = normal = without symptoms, 1 = symptoms, 2 = severe symptoms). The three coinfection groups (9–21 dpi) had significantly higher scores than the HP-PRRSV and PCV2 groups. Among the coinfection groups, the HP-PRRSV/PCV2 group showed the highest score (see Additional file 1: Table S1 for details). **(C)** The average daily weight gain in the HP-PRRSV/PCV2 group was negative (14–21 dpi), whereas the gains of the other groups were positive. Error bars show the standard deviations. * indicates significantly higher or lower values. * p < 0.05, ** p < 0.01; and n.s., not significant.

The average rectal temperatures (ART) (18–21 days postinoculation [dpi]) and average clinical scores (ACS; 17–21 dpi) of group 1 were significantly higher than those of group 2 (ART, *p* < 0.05; ACS, *p* < 0.05), group 3 (ART, *p* < 0.01; ACS, *p* < 0.05), and group 4 (ART, *p* < 0.01; ACS, *p* < 0.05; see Additional file 1: Table S1 for details). In group 1, three of the (≥ 40.5°C) at 6–24 dpi, all piglets developed severe wasting disease, and three died of severe respiratory distress at 21 dpi (14 days after PCV2 inoculation). The two remaining piglets in this group had severe dermatitis from 15 dpi to the end of the experiment. The mortality in group 1 was 60% (3/5), whereas it was 0% (0/5) in all other groups. The other HP-PRRSV-inoculated groups (groups 2–4) had less-severe clinical signs and all the piglets in these groups exhibited moderate wasting, dermatitis, and mild respiratory distress from 17 dpi (20 dpi in group 2) to the end of the experiment, with no deaths. In group 1, the average body weight of the piglets decreased over time, whereas it increased over time in the other groups (Figure 1 and Table 1).

**Table 1:** Frequency of selected clinical signs (n = 5 pigs per group)

**Group**	**Clinical signs**				
	**Depression**	**Erythema**	**Dyspnea**	**Conjunctivitis**	**Emaciation**
HP-PRRSV/PCV2 (group 1)	5/5	5/5	5/5	5/5	5/5
PCV2/HP-PRRSV (group 2)	2/5	2/5	3/5	4/5	4/5
HP-PRRSV+PCV2 (group 3)	5/5	2/5	3/5	5/5	5/5
HP-PRRSV (group 4)	1/5	0/5	2/5	5/5	2/5
PCV2 (group 5)	0/5	0/5	0/5	0/5	0/5
Control (group 6)	0/5	0/5	0/5	0/5	0/5

### Gross pathology

The macroscopic characteristics of the piglets are summarized in Table 2. Briefly, lesions were predominantly observed in the lymphatic system, lungs, and kidneys. Enlarged lymph nodes were observed in all piglets, except those in group 6. In each HP-PRRSV-inoculated group, the piglets developed lymph-node lesions that were usually characterized by moderate hemorrhage, whereas the hemorrhage in group 1 was severe. The pigs inoculated with concurrent or individual viruses had noncollapsed lungs with interstitial edema and enlarged interstitial tissue areas. Of the three piglets that died in group 1, all had swollen brown-colored kidneys and severe pulmonary venous congestion.

**Table 2:** Frequency of selected macroscopic lesions (n = 5 pigs per group)

**Lesions**	**Groups**					
	**HP-PRRSV/PCV2 (group 1)**	**PCV2/HP-PRRSV (group 2)**	**HP-PRRSV+PCV2 (group 3)**	**HP-PRRSV (group 4)**	**PCV2 (group 5)**	**Control (group 6)**
Lymph-node hemorrhage	5/5	3/5	3/5	2/5	1/5	0/5
Endocardial hemorrhage	4/5	3/5	1/5	2/5	0/5	0/5
Congestion of liver	2/5	1/5	2/5	2/5	0/5	0/5
Splenic infarction	3/5	1/5	1/5	0/5	0/5	0/5
Pulmonary congestion	5/5	2/5	3/5	2/5	1/5	0/5
Kidney gray spot	3/5	3/5	5/5	5/5	3/5	0/5
Brain edema	0/5	0/5	0/5	0/5	0/5	0/5
Duodenal mucous membrane swelling	2/5	2/5	2/5	2/5	0/5	0/5

### Histopathology

**Figure 2:**
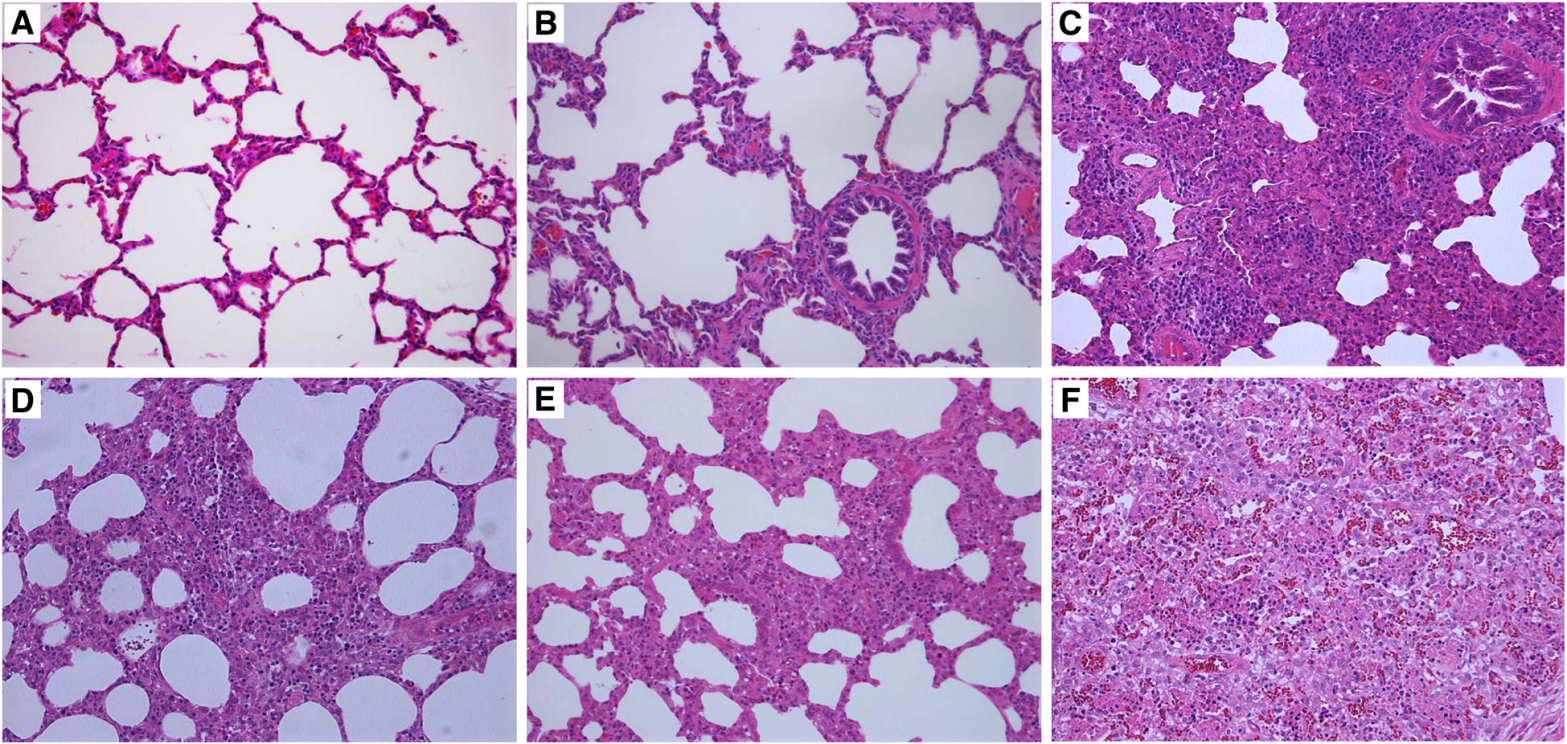
**Representative histopathological sections of lung from each infected group (200×).** Healthy control group **(A)** and PCV2 group **(B)**: no evident pathological changes. HP-PRRSV group **(C)**, HP-PRRSV+PCV2 group **(D)**, and PCV2/HP-PRRSV group **(E)** showed the same pathological changes: interstitial pneumonia and some stenosis or obstruction of the pulmonary alveoli. **(F)** The HP-PRRSV/PCV2 group had the most-severe pathological changes: severe congestion in small vessels and stenosis or obstruction of most pulmonary alveoli, which were full of necrotic and deciduous endothelial cells. Hematoxylin and eosin (HE) staining.

In the piglets inoculated with HP-PRRSV and/or PCV2 (groups 1–5), histopathological analyses revealed necrosis and/or lymphoid depletion in the lymph nodes, lymphocytic infiltration of the liver portal areas, thromboses in the small pulmonary blood vessels and alveolar capillaries, varying degrees of interstitial pneumonia (Figure 2), and plasma cell infiltrates in the duodenum, which are consistent with the results of a previous study [16] (Table 3). The kidneys of the PCV2-inoculated piglets (except those in groups 4 and 6) had mild perivascular infiltration of lymphocytes and macrophages. Intracytoplasmic inclusion bodies were observed in the renal macrophages of the piglets inoculated with PCV2 and HP-PRRSV (groups 1–3). The histological lesions were most severe in the HP-PRRSV/PCV2 group, followed by groups PCV2/HP-PRRSV and HP-PRRSV+PCV2, HP-PRRSV, and PCV2, when assessed according to a previously reported scoring system (data not shown) [16].

**Table 3:** **Histological lesions in the experimentally infected groups (*n *= 5)**

**Tissues lesion**	**Groups**					
	**HP-PRRSV/PCV2 (group 1)**	**PCV2/HP-PRRSV (group 2)**	**HP-PRRSV+PCV2 (group 3)**	**HP-PRRSV (group 4)**	**PCV2 (group 5)**	**Control (group 6)**
Lymphocytes infiltrating heart	3/5	2/5	3/5	2/5	0/5	0/5
Hepatic granular degeneration	1/5	0/5	4/5	3/5	0/5	0/5
Spleen lymphocyte depletion	4/5	1/5	0/5	0/5	1/5	0/5
Interstitial pneumonia	5/5	5/5	5/5	5/5	1/5	0/5
Tonsil lymphocyte depletion	2/5	1/5	0/5	0/5	0/5	0/5
Lymph-node lymphocyte depletion	4/5	3/5	1/5	2/5	1/5	0/5
Brain neuronal swelling	2/5	0/5	0/5	0/5	0/5	0/5
**Duodenal histiocytosis**	**5/5**	**3/5**	**1/5**	**3/5**	**1/5**	**0/5**

### HP-PRRSV viremia and distribution in tissues

**Figure 3:**
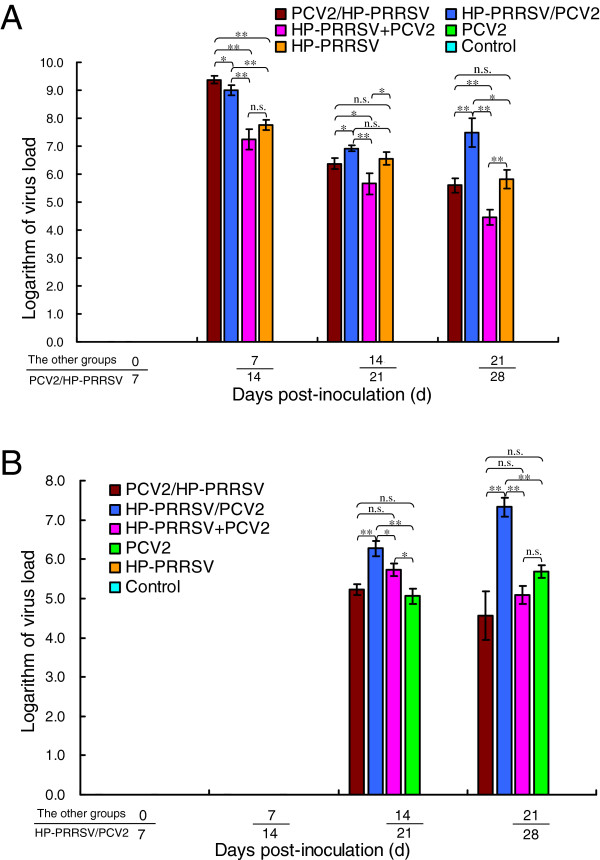
**Variation in HP-PRRSV and PCV2 nucleic acid loads in each experimentally infected group. (A)** The HP-PRRSV nucleic acid load in the HP-PRRSV/PCV2 group, in which viremia (in serum) was higher than in the other groups from 7 dpi and increased gradually thereafter. **(B)** PCV2 nucleic acid load in the HP-PRRSV/PCV2 group, in which viremia (in serum) was higher than in the other groups from 14 dpi, increased gradually, and peaked at 21 dpi. Error bars show the standard deviations. * indicates significantly higher or lower values. * p < 0.05, ** p < 0.01; and n.s., not significant.

HP-PRRSV viremia was detected in the serum samples from the HP-PRRSV-inoculated piglets from 3 dpi until 21 or 28 dpi, and in all postmortem tissues (Additional file 2: Table S2 and Additional file 3: Table S3). The highest levels of viral nucleic acids were detected in the sera (Figure 3A). The differences between the HP-PRRSV-inoculated groups were significant (*p* < 0.05) at 14 dpi (highest in group 2), 14 dpi, and 21 dpi (highest in group 1, lowest in group 3).

### HP-PRRSV antibodies

**Figure 4:**
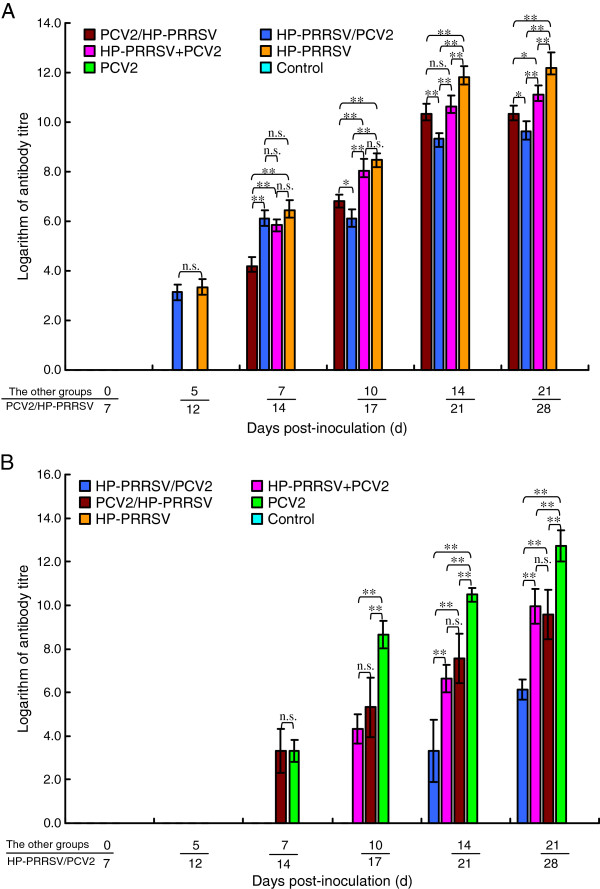
**Variations in levels of antibodies directed against HP-PRRSV and PCV2 in each experimentally infected group. (A)** The antibody titer was significantly lower in the HP-PRRSV/PCV2 group than in the other groups. **(B)** The titer of PCV2 antibodies was significantly lower in the HP-PRRSV/PCV2 group than in the other three infected groups. The antibody titer was significantly higher in the PCV2 group than in the three coinfection groups. Error bars show the standard deviations. * indicates significantly higher or lower values. * p < 0.05, ** p < 0.01; and n.s., not significant.

HP-PRRSV antibodies were detected in each group by 7 dpi (1:50), although antibodies were detected in group 4 at 5 dpi (1:50). The antibody titers increased with time, but the titer in group 1 was significantly lower than that in the other groups (*p* < 0.05), and this difference in titer gradually increased. The antibody titers of group 4 were significantly higher than those of the three coinfection groups (*p* < 0.05; Figure 4A).

### PCV2 viremia and distribution in tissues

PCV2 viremia was detected in group 1 at 10 dpi, peaked at 21 dpi, and continued until 28 dpi. In all other groups, PCV2 viremia peaked at 14 dpi, followed by a gradual reduction in the viral load (Additional file 2: Table S2 and Additional file 3: Table S3). Viral nucleic acids were detected in all postmortem tissues, and the lymphoid organs had the highest viral loads. The viral load in the serum samples from group 1 was significantly higher than those of the other groups (*p* < 0.01) and peaked at 21 dpi. The viral loads in the coinfection groups were significantly higher than that in group 5 (*p* < 0.05; Figure 3B).

### PCV2 antibodies

PCV2 antibodies were detected in groups 2, 5, 3, and 1 at 7 (1/5, 1:50), 7 (1/5, 1:50), 10 (2/5, 1:50), and 14 (1/5, 1:50) dpi, respectively, and increased gradually with time. The antibody titers in group 1 were significantly lower than those in the other three groups (*p* < 0.05) and the antibody titer in the group inoculated with PCV2 only was significantly higher than those in the three coinfection groups (*p* < 0.01; Figure 4B).

### Cytokines

**Figure 5:**
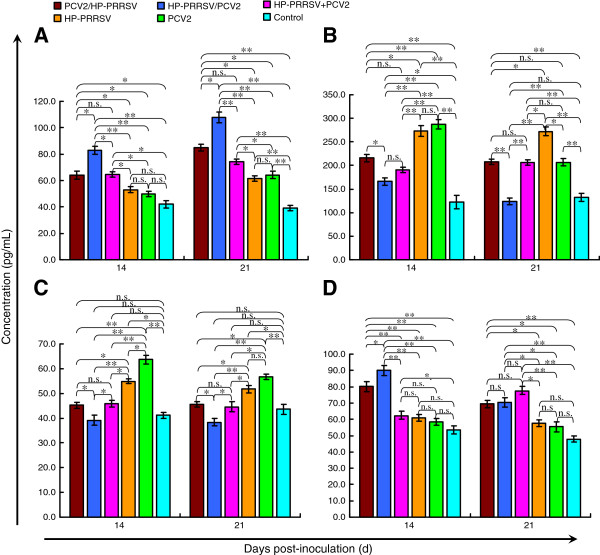
**Changes in cytokine levels in each experimentally infected group. (A)** The concentration of TNF-α was significantly higher in the HP-PRRSV/PCV2 group than in the other groups. The concentrations of IFN-γ **(B)** and GM-CSF **(C)** were lower in the coinfection groups, especially the HP-PRRSV/PCV2 group, than in the single-infection groups. **(D)** The concentration of IL-10 was higher in the coinfection groups, especially the HP-PRRSV/PCV2 group. Error bars show the standard deviations. * indicates significantly higher or lower values. * p < 0.05, ** p < 0.01; and n.s., not significant.

The levels of TNF-α in all the coinfection groups were elevated at 14 and 21 dpi, at which time the concentration of TNF-α (range, 83 ± 9.1–104 ± 8.6 pg/mL) was significantly higher in group 1 than in the other groups (*p* < 0.05 or 0.01; Figure 5A). In group 1, the IFN-γ concentrations were 124 ± 12.6 and 168 ± 13.4 pg/mL at 14 dpi and 21 dpi, respectively, and the GM-CSF concentrations were 38 ± 5.6 and 41 ± 7.6 pg/mL, respectively, whereas those in the other infected groups were significantly higher (*p* < 0.05 or 0.01; Figure 5B, C). The IL-10 level in group 1 (90 ± 7.4 pg/mL) at 14 dpi was higher than that in any other group (*p* < 0.05 or 0.01; Figure 5D). There were no significant differences in the assayed cytokine levels among groups 2–5, except in the IL-10 level in group 4 at 21 dpi.

### Flow cytometry

**Figure 6:**
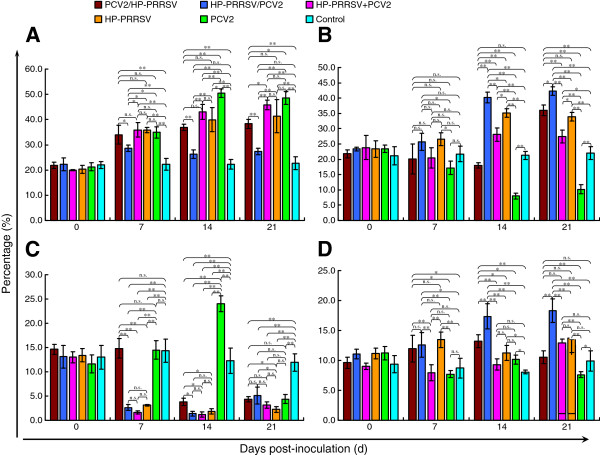
**Evolution of the immunocyte subpopulation in each experimentally infected group. (A)** The relative proportions of CD3^+^CD4^+^CD8^–^ cells to CD3^+^ cells increased continuously, with the lowest percentage in the HP-PRRSV/PCV2 group. **(B)** The proportions of CD3^+^CD4^--^CD8^+^ cells increased from 7 dpi, except in the PCV2 group, which showed a decline. **(C)** NK cells decreased in all groups over time. **(D)** The number of monocytes was greater in the HP-PRRSV/PCV2 group than in the other groups, and started to increase on day 7. Error bars show the standard deviations.* indicates significantly higher or lower values. * p < 0.05, ** p < 0.01; and n.s., not significant.

The ratio of CD3^+^/CD4^+^/CD8^–^ cells to CD3^+^ cells increased continuously from 7 dpi, with the lowest ratio in group 1 and the highest in group 5 (*p* < 0.05 or 0.01; Figure 6A). With the exception of group 5, in which a reduction occurred, the ratio of CD3^+^/CD4^–^/CD8^+^ cells to CD3^+^ cells increased from 7 dpi, and group 1 showed the most significant increase (*p* < 0.05 or 0.01; Figure 6B). NK cells decreased significantly in all infected groups (especially groups 1, 3, and 4) compared with the control group, except in groups 2 and 5 at 7 dpi (approximately equal to the control) and group 5 at 7 dpi (approximately equal to the control) and 14 dpi (significantly elevated) (*p* < 0.05 or 0.01; Figure 6C). The percentage of monocytes in group 1 started to increase at 7 dpi and was significantly higher than in the other infected groups from 14 dpi onward (*p* < 0.05 or 0.01; Figure 6D).

## Discussion

This study demonstrates that coinfection with HP-PRRSV and PCV2 can result in a more serious disease than infection with HP-PRRSV or PCV2 alone. A previous study showed that pigs inoculated with PRRSV before PCV2 can develop severe disease, with clinical manifestations and lesions characteristic of both PMWS and PRRS [17]. However, no previous study has compared sequential coinfection with HP-PRRSV and PCV2 or PRRSV and PCV2. The objective of this study was to systematically clarify the synergistic effects of HP-PRRSV and PCV2. As in other studies, the group infected with PCV2 alone showed no clinical signs, in contrast to the groups coinfected with HP-PRRSV and PCV2 [18], and the group infected with HP-PRRSV alone did not develop the clinical signs observed in the field (i.e., persistent high fever and high mortality). However, the presence of viral RNA/DNA and antibodies directed against PCV2 or PRRSV indicated that the piglets in groups 4 and 5 were successfully infected with HP-PRRSV and PCV2 respectively. An explanation of this phenomenon has been given previously [17], insofar as the different strains, inoculum methods, inoculation routes, doses, and source of pigs used are probably responsible for the severity of the clinical disease. However, based on the coinfections frequently reported in the field [13,14] and on the results of this study, it is more likely that coinfections and the interactions of the infective agents are involved in the induction of severe pathogenesis. This explanation better describes the single infection phenomena of groups 4 and 5. In the present study, piglets coinfected with HP-PRRSV and PCV2 had more-severe disease manifestations than those infected with HP-PRRSV or PCV2 alone, which is consistent with previous findings [17]. Like PRRSV, HP-PRRSV displays a synergistic effect with PCV2, which is consistent with the present experimental results. However, there was a large difference in pathogenicity among the three different coinfection combinations: HP-PRRSV/PCV2 had the strongest effect and caused the most-severe clinical signs, whereas only wasting and coarse hair were observed in the other groups.

The levels of HP-PRRSV and PCV2 nucleic acids were higher in the sera of pigs in the coinfected groups than in the sera of the singly infected groups, indicating that the two viruses synergistically affected replication. The sera of the piglets in the coinfection groups also contained sufficient numbers of viral copies (4.61 × 10^6^ copies/mL) to induce clinical signs of PMWS [19]. However, group 1 displayed the most-severe PMWS symptoms and had the highest viral load among the coinfection groups (group 1, 6.31 × 10^7^ copies/mL; group 2, 5.21 × 10^6^ copies/mL; and group 3, 5.43 × 10^6^ copies/mL). Consequently, more-severe lesions occurred in group 1, such as greater lymphocytic depletion in the lymph nodes, more-severe interstitial pneumonia, and more-severe clinical signs, and these lesions occurred in a larger number of piglets (3–5/5) than in the other coinfection groups (1–5/5 in group 3 and 0–5/5 in group 2; Tables 1, 2 and 3). These results are strong objective evidence of the synergistic effects of the viruses.

An analysis of postinfection antibody production showed that the antibody levels in the groups infected with the highly pathogenic viral combination HP-PRRSV/PCV2 were generally lower than those in the groups infected with the weakly pathogenic viral combinations PCV2/HP-PRRSV and HP-PRRSV+PCV2, and that the antibody levels correlated negatively with the viral load. Briefly, the pathogenicity of the coinfections was higher than that of the single infections, and the pathogenicity of the HP-PRRSV/PCV2 coinfection was higher than that of the PCV2/HP-PRRSV and HP-PRRSV+PCV2 coinfections. The main reason for these findings is that higher viral loads in the tissues led to more-severe damage to the immune system, which strongly inhibited antibody production. In contrast, infection with either of these viruses can suppress the immune response [1,20,21], which favors subsequent infections. Given the greater pathogenicity of HP-PRRSV, group 1 suffered from more severe disease than the other groups. It has also been reported that porcine alveolar macrophages (PAMs) that are infected with PCV2 in vitro can secrete large amounts of IFN-γ, thereby inhibiting subsequent infections of PAM by PRRSV [15]. This finding indirectly accounts for the higher levels of viral nucleic acids detected in group 1 (the HP-PRRSV/PCV2 group) than in those of group 2 (the PCV2/HP-PRRSV group) or group 3 (the HP-PRRSV+PCV2 group), and the more-severe organ lesions and lower antibody production in group 1. In contrast to a previous report [16], PCV2 nucleic acid was detected in all PCV2-inoculated pigs at 14 dpi, but not at 7 dpi, which was probably attributable to differences in the pigs used in the two studies.

A high TNF-α concentration can cause severe pathological damage [22], including hypersensitivity and severe bronchial constriction, which could have been an important cause of death in the experimental animals and might explain the highest mortality in group 1 (the HP-PRRSV/PCV2 group). The TNF-α levels in group 1 peaked at 14 and 21 dpi, and two piglets in this group died at 21 dpi. These TNF-α levels were significantly higher than those in the other groups. The levels of the positive immunoregulatory factors, GM-CSF and IFN-γ, were lower in the coinfection groups, especially in group 1. This suggests that the coinfection groups showed a weaker immune response than the single-infection groups, and that the response of group 1 was the weakest. IL-10, which plays a major role in the negative regulation of the immune response, was also highest in group 1, confirming that infection with HP-PRRSV before PCV2 led to a more severe disease state.

NK cells and monocytes are the major functional cells of the innate immune system, and a deficiency in these cells may significantly compromise the innate immune response in infected pigs. Subsets of immune cells, including CD4^+^ T and NK cells, were significantly reduced in the coinfection groups, especially in group 1, indicating that their adaptive and innate immune responses had been acutely suppressed. Nevertheless, at 14 dpi, group 1 had the highest level of CD8^+^ T cells, which mediate cellular immunity by killing target cells, although they can also indirectly cause serious tissue damage. Together, these results are evidence that the predominant immune response in the mid-anaphase of infection is cellular immunity. In group 1, the monocyte concentration increased markedly over time from 14 dpi to the end of the experiment, which was mistakenly considered to be a beneficial effect directed against the viral infection. However, an increase in monocytes and a reduction in CD25^+^ cells are characteristic of PMWS in the field [23]. The piglets in group 1 developed the most-severe infections, even though the ratio of CD25^+^ cells did not change, which is consistent with the clinical data and the micro- and macropathological changes observed.

In summary, the pathogenesis of HP-PRRSV and PCV2 is synergistic, especially when an animal is infected with HP-PRRSV before PCV2. As reported previously, PCV2 can be detected in normal healthy pigs [24-26], suggesting that PCV2 can hide in the body until another pathogen infects the host. This has been confirmed by Krakowka [27], who concluded that an immunogen can trigger PMWS in pigs infected with PCV2. The usual explanation is that the replication of circoviral DNA is dependent upon host cell enzymes expressed during the S-phase of the cell cycle, when the cell stimulated with a mitogen. We hypothesized that if this stimulation occurs before PCV2 infection, PCV2 would be replicated rapidly and abundantly, leading to a severe disease state. Furthermore, if the stimulus is a pathogen that causes immunosuppression, such as PRRSV [28], the disease will be even more severe. PRRSV infection can cause hyperplasia of the lymph nodes, particularly at 7–10 dpi [29], which suggests that PRRSV can be considered a mitogen of immunocytes. This has been confirmed by Rovira et al. [17]. In the HP-PRRSV/PCV2 group in the present study, the HP-PRRSV infection created the conditions for PCV2 replication in the cells, after which the pathopoiesis of HP-PRRSV and PCV2 combined into one unit, with an amplification effect. This is supported by the finding of clinical symptoms, average daily weight gains, gross lesions, pathology, antibody yield, viral loads in the sera, and specific cytokine and immunocyte subgroups. In the simultaneous coinfection group and the PCV2/HP-PRRSV group, competitive inhibition may have occurred as these two viruses vied for resources, or the optimal time for PCV2 replication after infection did not coincide with the optimal conditions induced by the HP-PRRSV infection.

It is widely known that a weak innate immune response results in a weak adaptive immune response. Monocytes/macrophages and the cytokines they secrete play crucial roles in initiating the adaptive immune response. Interestingly, PRRSV and PCV2 can replicate in monocyte/macrophage-lineage cells, including alveolar macrophages, in the lymph nodes and tissues [30,31]. TNF-α and IL-10 are mainly secreted by activated monocytes/macrophages, and PRRSV infection can significantly enhance the expression of these two kinds of cytokines [32,33]. Therefore, in group 1, the earlier HP-PRRSV infection primed the expression of TNF-α and IL-10 in the first seven days, which was then enhanced by the PCV2 infection. With the immunosuppression of IL-10, the expression of other cytokines was inhibited, as were the immunocyte subgroups whose proliferation is mediated by many cytokines responsible for the positive regulation of the immune response. Therefore, the proportions of CD4^+^ and NK cells decreased. Interestingly, the numbers of CD8^+^ cells increased. Further research is required to understand why.

## Conclusions

In this study, the effects of sequential HP-PRRSV and PCV2 infections were investigated, and the synergistic pathogenesis of the two pathogenic viruses was analyzed comprehensively. The data suggest that an earlier HP-PRRSV infection and a subsequent PCV2 infection can increase the severity of the disease. Our findings provide a foundation for further research to clarify the mechanism underlying the synergistic pathogenicity of HP-PRRSV and PCV2.

## Materials and methods

### Cell lines and viruses

Two cell lines, Marc-145 (derived from the African green monkey kidney cell line) and porcine kidney (PK), were infected with a highly pathogenic mutated strain of PRRSV (HP-PRRSV strain HBR) or PCV2 (PCV2b, YJ strain; GenBank accession no. HM038032) isolated at the Harbin Veterinary Research Institute (Chinese Academy of Agricultural Sciences, Harbin, China). The experiments with infected animals were performed with viruses from the 10th passage of these two viruses in culture and the viral infectious dose was adjusted to 10^4.5^ 50% tissue culture infective doses (TCID_50_)/mL.

### Experimental design

Thirty 35-day-old healthy, conventional, mixed-sex Yorkshire piglets from four different litters were used in this study. All the piglets were seronegative for PRRSV, PCV2, porcine parvovirus, pseudorabies virus, and classic swine fever virus according to enzyme-linked immunosorbent assays (ELISAs), and were free of viral nucleic acids according to reverse transcription–polymerase chain reaction (RT–PCR) and PCR analyses [34-38]. The piglets were housed in a physical containment level 2 laboratory at 25°C throughout the experiment. This study was approved by the Harbin Veterinary Research Institute, Chinese Academy of Agricultural Sciences (approval number Heilongjiang-SYXK-2006-032).

The piglets were randomly divided into six groups of five piglets each: HP-PRRSV/PCV2 (group 1), in which HP-PRRSV was inoculated first and PCV2 seven days later; PCV2/HP-PRRSV (group 2), in which PCV2 was inoculated first and HP-PRRSV seven days later; HP-PRRSV+PCV2 (group 3), in which the viruses were inoculated concurrently; HP-PRRSV only (group 4); PCV2 only (group 5); and uninfected pigs (group 6), as the control. These piglets were managed according to a previous study [16]. Briefly, the piglets were housed in separate isolation rooms with negative pressure ventilation. Workers had had no other contact with pigs for 12 h and showered and changed their clothes before entry into the isolation rooms. Before entering, all personnel changed into coveralls, hairnets, face masks, gloves, and disposable boots and used a foot bath. The flow of people was unidirectional from the uninoculated rooms to the inoculated rooms, and separate equipment was supplied to each room. Each pig was inoculated with 1 mL of virus intranasally and 1 mL of virus intramuscularly in the neck, a total of 2 mL of inoculum per pig per virus. The HP-PRRSV and PCV2 inocula were given separately, not mixed, when install/infect into another nostril/infection site one after the other, which was applied to both virus. Rectal temperatures and clinical symptoms were recorded daily and body weights were measured weekly. Blood samples were collected at 0, 3, 5, 7, 10, 14, and 21 dpi for flow cytometry and serum separation.

The pigs in the simultaneous coinfection group (group 3) and the single infection groups (groups 4 and 5) were killed at 21 dpi, whereas the piglets in the sequential coinfection groups and control group (groups 1, 2, and 6) were killed 28 days after the first inoculation. Heart, liver, spleen, lung, kidney, brain, duodenal, and lymph-node tissues were harvested for histopathological analysis and nucleic acid detection. To test for the presence of viral nucleic acids, the tissues were homogenized and subjected to three freeze/thaw cycles, after which the supernatants were collected. The severity of the clinical signs was scored and evaluated using cumulative scores, as described by Opriessnig et al. (2004) [39] (Figure 1).

### HP-PRRSV RNA detection

Total RNA was extracted from serum and tissue samples using TRIzol Reagent (BioFlux Corp., Tokyo, Japan) and stored at −80°C. Viral RNA was detected in the serum and tissues, and was also quantified in the serum by real-time RT–PCR using a Rotor Gene 3000 Real-Time PCR instrument (Corbett Robotics Pty., Ltd, Brisbane, Australia), according to the manufacturer’s instructions [40].

### PRRSV antibody detection

PRRSV antibodies were detected in sera using the immunoperoxidase monolayer assay (IPMA) [41].

### PCV2 DNA detection

Viral DNA was isolated from serum and tissue samples with proteinase K digestion (Takara Bio, Inc., Shinga, Japan) and phenol–chloroform–isoamyl alcohol extraction. Viral DNA was detected in the sera and tissues and was also quantified in the serum samples with a quantitative PCR method, as reported by Opriessnig et al. (2003) [42].

### PCV2 antibody detection

PCV2 antibodies were detected in the serum samples using the IPMA method [43].

### Cytokine detection

Levels of porcine interferon γ (IFN-γ), tumor necrosis factor α (TNF-α), interleukin 10 (IL-10), and granulocyte macrophage-colony stimulating factor (GM-CSF) were measured in the serum samples using commercial ELISA kits (Market, USA).

### Flow cytometry

Cluster of differentiation (CD)3^+^/CD4^+^/CD8^–^, CD3^+^/CD4^+^/CD8^+^, natural killer (NK), and CD3^–^/CD4^–^/CD8^+^ cells in the peripheral blood were quantified by three-color flow cytometry using a fluorescence-activated cell sorter (FACSAria flow cytometer, Becton Dickinson & Company, Franklin Lakes, NJ, USA). The monoclonal antibodies used in this study were mouse anti-pig CD3–spectral red (SPRD), anti-pig CD4–fluorescein isothiocyanate (FITC), and anti-pig CD8–phycoerythrin (PE) (SouthernBiotech, Birmingham, AL, USA). Monocytes (SWC3a^+^/SSC^low/–^)* were quantified by one-color flow cytometry using a mouse anti-pig SWC3a–PE monoclonal antibody (Becton Dickinson & Company). *SWC3a (swine workshop cluster number 3a) is expressed on the cell membranes of monocytes, macrophages, and granulocytes; SSC (side scatter) indicates cellular granularity.

### Statistical analysis

Statistical analyses were performed with SPSS (PASW Statistics, Chicago, IL, USA) and Microsoft Excel software (Microsoft Corp., Redmond, WA, USA). All data were averaged and the differences in mean values between each pair of groups were analyzed with multivariate analysis of variance using Tukey’s honestly significant difference (HSD) post hoc test.

## Competing interests

None of the authors of this paper has a financial or personal relationship with other people or organizations that could inappropriately influence or bias the content of the paper.

## Authors’ contributions

Puihu Fan and Yanwu Wei performed all the experiments, participated in the study design, and drafted the manuscript. Longjun Guo, Hongli Wu, and Liping Huang performed the immunoassays. Jianbo Liu dissected some of the experimental piglets at the end of the experiment. Changming Liu conceived the study, participated in its design, and helped to draft the manuscript. All the authors have read and approved the final manuscript.

## Supplementary Material

Additional file 1: Table S1 Comparison of average rectal temperatures and clinical sign scores of with each group on days postinoculation. Click here for file

Additional file 2: Table S2 Detection of HP-PRRSV and PCV2 viremia in each infected group by RT–PCR/PCR (days postinoculation). Click here for file

Additional file 3: Table S3 Detection of HP-PRRSV and PCV2 in each organ of each infected group by RT–PCR/PCR. Click here for file
