# Supplementary material for: Synergistic effects of sequential infection with highly pathogenic porcine reproductive and respiratory syndrome virus and porcine circovirus type 2
Source: Virol J. 2013 Aug 26;10:265. doi: 10.1186/1743-422X-10-265 (PMC3847690; doi:10.1186/1743-422X-10-265)
Supplement: Additional file 1: Table S1 — Comparison of average rectal temperatures and clinical sign scores of with each group on days postinoculation. [file 1743-422X-10-265-S1.doc]

**Additional file 1 –Table S1.**

Comparison of average rectal temperatures and clinical sign scores of with each group on days postinoculation.

| DPI | Groups | Average rectal temperature | Significant difference level | | Groups | Clinical sign score | Significant difference level | |
| --- | --- | --- | --- | --- | --- | --- | --- | --- |
|  |  |  | *p*<0.05 | *p*<0.01 |  |  | *p*<0.05 | *p*<0.01 |
| 0 | HP-PRRSV | 39.3±0.15 | a | A | HP-PRRSV/PCV2 | 0±0 | a | A |
|  | HP-PRRSV+PCV2 | 39.2±0.11 | a | A | PCV2/HP-PRRSV | 0±0 | a | A |
|  | HP-PRRSV/PCV2 | 39.2±0.13 | a | A | HP-PRRSV+PCV2 | 0±0 | a | A |
|  | PCV2/HP-PRRSV | 39.2±0.12 | a | A | HP-PRRSV | 0±0 | a | A |
|  | PCV2 | 39.2±0.10 | a | A | PCV2 | 0±0 | a | A |
|  | Control | 39.1±0.11 | a | A | Control | 0±0 | a | A |
| 1 | HP-PRRSV/PCV2 | 39.5±0.13 | a | A | HP-PRRSV/PCV2 | 0±0 | a | A |
|  | HP-PRRSV+PCV2 | 39.5±0.12 | ab | A | PCV2/HP-PRRSV | 0±0 | a | A |
|  | HP-PRRSV | 39.2±0.32 | bc | A | HP-PRRSV+PCV2 | 0±0 | a | A |
|  | PCV2 | 39.2±0.13 | c | A | HP-PRRSV | 0±0 | a | A |
|  | PCV2/HP-PRRSV | 39.1±0.12 | c | A | PCV2 | 0±0 | a | A |
|  | Control | 39.1±0.11 | c | A | Control | 0±0 | a | A |
| 2 | HP-PRRSV/PCV2 | 40.4±0.23 | a | A | HP-PRRSV/PCV2 | 0.2±0.52 | a | A |
|  | HP-PRRSV+PCV2 | 40.2±0.19 | ab | AB | PCV2/HP-PRRSV | 0±0 | a | A |
|  | HP-PRRSV | 39.6±0.28 | abc | ABC | HP-PRRSV+PCV2 | 0±0 | a | A |
|  | PCV2 | 39.4±0.10 | d | C | HP-PRRSV | 0±0 | a | A |
|  | PCV2/HP-PRRSV | 39.3±0.13 | d | C | PCV2 | 0±0 | a | A |
|  | Control | 39.1±0.14 | d | C | Control | 0±0 | a | A |
| 3 | HP-PRRSV/PCV2 | 39.9±0.15 | a | A | HP-PRRSV/PCV2 | 0.4±0.43 | a | A |
|  | HP-PRRSV+PCV2 | 39.8±0.12 | ab | AB | PCV2/HP-PRRSV | 0±0 | a | A |
|  | HP-PRRSV | 39.7±0.15 | abc | ABC | HP-PRRSV+PCV2 | 0±0 | a | A |
|  | PCV2 | 39.4±0.13 | d | ABC | HP-PRRSV | 0±0 | a | A |
|  | PCV2/HP-PRRSV | 39.1±0.23 | d | C | PCV2 | 0±0 | a | A |
|  | Control | 39.1±0.13 | d | C | Control | 0±0 | a | A |
| 4 | HP-PRRSV+PCV2 | 39.7±0.11 | a | A | HP-PRRSV/PCV2 | 0±0 | a | A |
|  | HP-PRRSV/PCV2 | 39.6±0.12 | ab | A | PCV2/HP-PRRSV | 0±0 | a | A |
|  | PCV2/HP-PRRSV | 39.5±0.14 | abc | A | HP-PRRSV+PCV2 | 0±0 | a | A |
|  | HP-PRRSV | 39.5±0.13 | abc | A | HP-PRRSV | 0±0 | a | A |
|  | PCV2 | 39.4±0.11 | abc | A | PCV2 | 0±0 | a | A |
|  | Control | 39.1±0.10 | c | A | Control | 0±0 | a | A |
| 5 | HP-PRRSV+PCV2 | 39.8±0.12 | a | A | HP-PRRSV/PCV2 | 0.4±0.42 | a | A |
|  | HP-PRRSV/PCV2 | 39.8±0.13 | ab | AB | HP-PRRSV | 0.3±0.51 | a | A |
|  | HP-PRRSV | 39.8±0.12 | abc | ABC | PCV2/HP-PRRSV | 0±0 | a | A |
|  | PCV2/HP-PRRSV | 39.6±0.13 | abcd | ABC | HP-PRRSV+PCV2 | 0±0 | a | A |
|  | PCV2 | 39.5±0.11 | abcde | ABC | PCV2 | 0±0 | a | A |
|  | Control | 39.2±0.14 | f | C | Control | 0±0 | a | A |
| 6 | HP-PRRSV/PCV2 | 40.4±0.15 | a | A | HP-PRRSV | 0.4±0.4 | a | A |
|  | HP-PRRSV+PCV2 | 40.3±0.14 | ab | AB | HP-PRRSV/PCV2 | 0.2±0.45 | a | A |
|  | HP-PRRSV | 40.1±0.15 | abc | ABC | HP-PRRSV+PCV2 | 0.2±0.52 | a | A |
|  | PCV2/HP-PRRSV | 39.4±0.13 | d | D | PCV2/HP-PRRSV | 0±0 | a | A |
|  | PCV2 | 39.4±0.14 | d | D | PCV2 | 0±0 | a | A |
|  | Control | 39.2±0.11 | d | D | Control | 0±0 | a | A |
| 7 | HP-PRRSV/PCV2 | 40.3±0.13 | a | A | HP-PRRSV | 1.8±0.54 | a | A |
|  | HP-PRRSV+PCV2 | 40.1±0.12 | ab | AB | HP-PRRSV+PCV2 | 1.4±0.48 | a | A |
|  | HP-PRRSV | 39.9±0.15 | abc | ABC | PCV2 | 0.8±0.43 | a | A |
|  | PCV2/HP-PRRSV | 39.5±0.14 | d | D | HP-PRRSV/PCV2 | 0.6±0.51 | a | A |
|  | PCV2 | 39.4±0.12 | d | D | PCV2/HP-PRRSV | 0±0 | a | A |
|  | Control | 39.2±0.11 | d | D | Control | 0±0 | a | A |
| 8 | HP-PRRSV/PCV2 | 40.0±0.12 | a | A | HP-PRRSV+PCV2 | 1.8±0.43 | a | A |
|  | HP-PRRSV+PCV2 | 39.8±0.13 | ab | AB | HP-PRRSV | 1.8±0.41 | a | A |
|  | HP-PRRSV | 39.7±0.14 | abc | AB | HP-PRRSV/PCV2 | 1.4±0.40 | a | A |
|  | PCV2/HP-PRRSV | 39.5±0.13 | d | B | PCV2 | 1.2±0.51 | a | A |
|  | PCV2 | 39.4±0.13 | de | B | PCV2/HP-PRRSV | 0±0 | b | B |
|  | Control | 39.1±0.11 | f | B | Control | 0±0 | b | B |
| 9 | HP-PRRSV+PCV2 | 40.5±0.13 | a | A | HP-PRRSV | 2.6±0.46 | a | A |
|  | HP-PRRSV/PCV2 | 40.3±0.12 | ab | AB | HP-PRRSV+PCV2 | 2.6±0.45 | ab | A |
|  | HP-PRRSV | 40.1±0.14 | abc | ABC | HP-PRRSV/PCV2 | 1.6±0.42 | abc | A |
|  | PCV2/HP-PRRSV | 39.8±0.12 | d | CD | PCV2 | 1.2±0.52 | cd | A |
|  | PCV2 | 39.5±0.11 | e | D | PCV2/HP-PRRSV | 0.6±0.53 | cde | A |
|  | Control | 39.3±0.11 | e | D | Control | 0±0 | f | B |
| 10 | HP-PRRSV+PCV2 | 40.6±0.12 | a | A | HP-PRRSV+PCV2 | 3±0.45 | a | A |
|  | HP-PRRSV/PCV2 | 40.5±0.11 | ab | AB | HP-PRRSV | 2.8±0.56 | ab | A |
|  | PCV2/HP-PRRSV | 40.3±0.14 | abc | ABC | HP-PRRSV/PCV2 | 2±0.43 | abc | A |
|  | HP-PRRSV | 40.0±0.13 | bcd | ABCD | PCV2 | 1.6±0.56 | abcd | A |
|  | PCV2 | 39.5±0.12 | e | E | PCV2/HP-PRRSV | 1±0.54 | e | A |
|  | Control | 39.2±0.10 | f | E | Control | 0±0 | f | B |
| 11 | HP-PRRSV/PCV2 | 40.6±0.14 | a | A | HP-PRRSV+PCV2 | 3.6±0.62 | a | A |
|  | HP-PRRSV+PCV2 | 40.4±0.14 | ab | AB | HP-PRRSV | 2.8±0.61 | ab | A |
|  | HP-PRRSV | 40.2±0.13 | bc | ABC | HP-PRRSV/PCV2 | 2.2±0.51 | abc | A |
|  | PCV2/HP-PRRSV | 40.0±0.12 | bcd | ABCD | PCV2 | 1.4±0.43 | abcd | A |
|  | PCV2 | 39.6±0.10 | e | E | PCV2/HP-PRRSV | 0.6±0.52 | e | A |
|  | Control | 39.2±0.10 | f | E | Control | 0±0 | f | B |
| 12 | HP-PRRSV+PCV2 | 40.4±0.14 | a | A | HP-PRRSV+PCV2 | 4±0.46 | a | A |
|  | HP-PRRSV/PCV2 | 40.3±0.11 | ab | AB | HP-PRRSV/PCV2 | 2.8±0.54 | b | AB |
|  | PCV2/HP-PRRSV | 40.2±0.13 | abc | ABC | HP-PRRSV | 2.6±0.54 | bc | ABC |
|  | HP-PRRSV | 40.1±0.14 | abcd | ABCD | PCV2 | 1.4±0.46 | d | BCD |
|  | PCV2 | 39.8±0.11 | e | E | PCV2/HP-PRRSV | 0.6±0.52 | de | BCDE |
|  | Control | 39.3±0.12 | f | F | Control | 0±0 | f | F |
| 13 | PCV2/HP-PRRSV | 40.5±0.14 | a | A | HP-PRRSV+PCV2 | 4.2±0.54 | a | A |
|  | HP-PRRSV/PCV2 | 40.3±0.13 | ab | AB | HP-PRRSV/PCV2 | 2.8±0.46 | b | AB |
|  | HP-PRRSV | 40.1±0.15 | abc | ABC | HP-PRRSV | 2±0.45 | bc | ABC |
|  | HP-PRRSV+PCV2 | 39.8±0.16 | cd | CD | PCV2 | 1.4±0.53 | bcd | BCD |
|  | PCV2 | 39.7±0.13 | de | DE | PCV2/HP-PRRSV | 0.8±0.52 | de | BCDE |
|  | Control | 39.1±0.12 | f | F | Control | 0±0 | f | F |
| 14 | PCV2/HP-PRRSV | 40.3±0.13 | a | A | HP-PRRSV+PCV2 | 6.2±0.56 | a | A |
|  | HP-PRRSV/PCV2 | 40.3±0.12 | ab | AB | HP-PRRSV/PCV2 | 3.2±0.53 | b | B |
|  | HP-PRRSV | 40.3±0.11 | abc | ABC | HP-PRRSV | 3±0.46 | bc | BC |
|  | HP-PRRSV+PCV2 | 39.9±0.12 | abcd | ABCD | PCV2/HP-PRRSV | 1.8±0.52 | d | BCD |
|  | PCV2 | 39.5±0.11 | de | DE | PCV2 | 1.6±0.53 | de | BCDE |
|  | Control | 39.2±0.13 | f | E | Control | 0±0 | f | F |
| 15 | PCV2/HP-PRRSV | 40.2±0.14 | a | A | HP-PRRSV+PCV2 | 6±0.41 | a | A |
|  | HP-PRRSV | 40.2±0.15 | ab | AB | HP-PRRSV/PCV2 | 5±0.46 | ab | AB |
|  | HP-PRRSV/PCV2 | 40.1±0.14 | abc | ABC | HP-PRRSV | 4.6±0.52 | abc | ABC |
|  | HP-PRRSV+PCV2 | 39.8±0.13 | d | ABCD | PCV2/HP-PRRSV | 1.8±0.52 | d | D |
|  | PCV2 | 39.6±0.13 | de | DE | PCV2 | 1.6±0.54 | de | DE |
|  | Control | 39.3±0.11 | f | E | Control | 0±0 | f | F |
| 16 | PCV2/HP-PRRSV | 40.2±0.13 | a | A | HP-PRRSV/PCV2 | 5.4±0.43 | a | A |
|  | HP-PRRSV/PCV2 | 40.1±0.13 | ab | AB | HP-PRRSV+PCV2 | 5.2±0.48 | ab | AB |
|  | HP-PRRSV | 40.1±0.12 | abc | ABC | HP-PRRSV | 3.8±0.50 | c | ABC |
|  | PCV2 | 39.6±0.12 | d | D | PCV2/HP-PRRSV | 3.2±0.41 | cd | CD |
|  | HP-PRRSV+PCV2 | 39.6±0.13 | de | DE | PCV2 | 1.6±0.53 | e | CDE |
|  | Control | 39.2±0.10 | f | F | Control | 0±0 | f | F |
| 17 | PCV2/HP-PRRSV | 40.3±0.13 | a | A | HP-PRRSV/PCV2 | 5.8±0.53 | a | A |
|  | HP-PRRSV/PCV2 | 40.1±0.13 | ab | AB | HP-PRRSV | 4.6±0.51 | b | AB |
|  | HP-PRRSV | 40.1±0.14 | abc | ABC | HP-PRRSV+PCV2 | 4.4±0.32 | bc | ABC |
|  | PCV2 | 39.5±0.12 | d | D | PCV2/HP-PRRSV | 3.2±0.56 | d | CD |
|  | HP-PRRSV+PCV2 | 39.4±0.11 | de | D | PCV2 | 1.4±0.52 | e | E |
|  | Control | 39.2±0.12 | f | D | Control | 0±0 | f | F |
| 18 | HP-PRRSV/PCV2 | 40.6±0.14 | a | A | HP-PRRSV/PCV2 | 6.2±0.53 | a | A |
|  | PCV2/HP-PRRSV | 40.1±0.11 | b | B | HP-PRRSV+PCV2 | 4.2±0.46 | b | B |
|  | HP-PRRSV | 39.7±0.13 | c | C | PCV2/HP-PRRSV | 4±0.46 | bc | BC |
|  | PCV2 | 39.3±0.12 | d | D | HP-PRRSV | 3.8±0.54 | bcd | BCD |
|  | HP-PRRSV+PCV2 | 39.3±0.14 | d | D | PCV2 | 1.4±0.53 | e | E |
|  | Control | 39.1±0.11 | d | D | Control | 0±0 | f | F |
| 19 | HP-PRRSV/PCV2 | 40.3±0.14 | a | A | HP-PRRSV/PCV2 | 5±0.50 | a | A |
|  | PCV2/HP-PRRSV | 40.1±0.13 | ab | AB | HP-PRRSV | 4±0.48 | b | AB |
|  | HP-PRRSV | 39.4±0.12 | c | C | PCV2/HP-PRRSV | 3.8±0.45 | bc | ABC |
|  | PCV2 | 39.3±0.12 | cd | C | HP-PRRSV+PCV2 | 3.8±0.46 | bcd | ABCD |
|  | Control | 39.2±0.12 | cd | C | PCV2 | 1.4±0.43 | e | E |
|  | HP-PRRSV+PCV2 | 39.0±0.15 | d | C | Control | 0±0 | f | F |
| 20 | HP-PRRSV/PCV2 | 40.3±0.13 | a | A | HP-PRRSV/PCV2 | 5.8±0.43 | a | A |
|  | PCV2/HP-PRRSV | 40.0±0.12 | b | AB | PCV2/HP-PRRSV | 4.4±0.46 | b | AB |
|  | HP-PRRSV | 39.6±0.13 | c | C | HP-PRRSV | 4.2±0.47 | bc | ABC |
|  | HP-PRRSV+PCV2 | 39.4±0.14 | cd | C | HP-PRRSV+PCV2 | 3.8±0.52 | bcd | ABCD |
|  | PCV2 | 39.3±0.14 | cde | C | PCV2 | 1±0.57 | e | E |
|  | Control | 38.9±0.11 | f | C | Control | 0±0 | f | F |
| 21 | HP-PRRSV/PCV2 | 40.8±0.12 | a | A | HP-PRRSV/PCV2 | 6.8±0.51 | a | A |
|  | PCV2/HP-PRRSV | 40.1±0.13 | b | B | PCV2/HP-PRRSV | 5±0.46 | b | B |
|  | HP-PRRSV | 39.6±0.12 | c | C | HP-PRRSV | 4.6±0.52 | bc | BC |
|  | HP-PRRSV+PCV2 | 39.5±0.13 | cd | CD | HP-PRRSV+PCV2 | 3.8±0.51 | bcd | BCD |
|  | PCV2 | 39.2±0.12 | e | E | PCV2 | 1±0.53 | e | E |
|  | Control | 39.1±0.10 | e | E | Control | 0±0 | f | F |
