# Supplementary material for: Synergistic effects of sequential infection with highly pathogenic porcine reproductive and respiratory syndrome virus and porcine circovirus type 2
Source: Virol J. 2013 Aug 26;10:265. doi: 10.1186/1743-422X-10-265 (PMC3847690; doi:10.1186/1743-422X-10-265)
Supplement: Additional file 2: Table S2 — Detection of HP-PRRSV and PCV2 viremia in each infected group by RT–PCR/PCR (days postinoculation). [file 1743-422X-10-265-S2.doc]

**Additional file 2 –Table S2.**

Detection of HP-PRRSV and PCV2 viremia in each infected group by RT–PCR/PCR (days postinoculation).

| Group | No. | Days postinoculation | | | | | | | | |
| --- | --- | --- | --- | --- | --- | --- | --- | --- | --- | --- |
|  |  | 0 d | 3 d | 5 d | 7 d | 10 d | 14 d | 17 d | 21d | 28 d |
| HP-PRRSV/PCV2  (group 1) | 12 | －/－ | 3＋/－ | 4＋/－ | 4＋/－ | 4＋/－ | 4＋/－ | 4＋/＋ | 4＋/4＋ | 4＋/4＋ |
|  | 13 | －/－ | 4＋/－ | 4＋/－ | 4＋/－ | 4＋/－ | 4＋/－ | 4＋/＋ | 4＋/4＋ | 4＋/4＋ |
|  | 16 | －/－ | 4＋/－ | 4＋/－ | 4＋/－ | 4＋/－ | 4＋/－ | 4＋/＋ | 4＋/4＋ | 4＋/4＋ |
|  | 17 | －/－ | 4＋/－ | 4＋/－ | 4＋/－ | 4＋/－ | 4＋/－ | 4＋/＋ | 4＋/4＋ | Died/Died |
|  | 18 | －/－ | 4＋/－ | 4＋/－ | 4＋/－ | 4＋/－ | 4＋/－ | 4＋/＋ | 4＋/4＋ | Died/Died |
| PCV2/HP-PRRSPV  (group 2) | 1 | －/－ | －/－ | －/－ | －/－ | 2＋/－ | 4＋/2＋ | ＋/3＋ | 4＋/4＋ | ＋/3＋ |
|  | 2 | －/－ | －/－ | －/－ | －/－ | 2＋/－ | 4＋/2＋ | 4＋/3＋ | ＋/4＋ | ＋/2＋ |
|  | 3 | －/－ | －/－ | －/－ | －/－ | 3＋/－ | 4＋/4＋ | 4＋/4＋ | 4＋/4＋ | ＋/2＋ |
|  | 4 | －/－ | －/－ | －/－ | －/－ | 3＋/－ | 4＋/2＋ | 4＋/2＋ | 4＋/4＋ | ＋/1＋ |
|  | 5 | －/－ | －/－ | －/－ | －/－ | 3＋/－ | 4＋/3＋ | 4＋/3＋ | 4＋/4＋ | ＋/2＋ |
| HP-PRRSV+PCV2  (group 3) | 6 | －/－ | 4＋/－ | 4＋/－ | 4＋/－ | 4＋/－ | 4＋/3＋ | ND/ND | 2＋/2＋ | ND/ND |
|  | 8 | －/－ | 4＋/－ | 4＋/－ | 4＋/－ | 4＋/－ | 4＋/2＋ | ND/ND | 2＋/2＋ | ND/ND |
|  | 9 | －/－ | 4＋/－ | 3＋/－ | 4＋/－ | 4＋/－ | ＋/2＋ | ND/ND | －/3＋ | ND/ND |
|  | 10 | －/－ | 4＋/－ | 2＋/－ | 4＋/－ | 4＋/－ | 4＋/3＋ | ND/ND | 2＋/3＋ | ND/ND |
|  | 11 | －/－ | 4＋/－ | 2＋/－ | 4＋/－ | 4＋/－ | 4＋/2＋ | ND/ND | 2＋/＋ | ND/ND |
| HP-PRRSV  (group 4) | 7 | －/－ | 4＋/－ | 4＋/－ | 3＋/－ | 3＋/－ | 3＋/－ | ND/ND | 2＋/－ | ND/ND |
|  | 14 | －/－ | 4＋/－ | 4＋/－ | 3＋/－ | 3＋/－ | 3＋/－ | ND/ND | 2＋/－ | ND/ND |
|  | 15 | －/－ | 4＋/－ | 4＋/－ | 3＋/－ | 3＋/－ | 3＋/－ | ND/ND | 2＋/－ | ND/ND |
|  | 19 | －/－ | 4＋/－ | 4＋/－ | 3＋/－ | 3＋/－ | 3＋/－ | ND/ND | 2＋/－ | ND/ND |
|  | 20 | －/－ | 4＋/－ | 4＋/－ | 3＋/－ | 3＋/－ | 3＋/－ | ND/ND | 2＋/－ | ND/ND |
| PCV2 (group 5) | 21 | －/－ | －/－ | －/－ | －/－ | －/－ | －/3＋ | ND/ND | －/4＋ | ND/ND |
|  | 22 | －/－ | －/－ | －/－ | －/－ | －/－ | －/2＋ | ND/ND | －/3＋ | ND/ND |
|  | 23 | －/－ | －/－ | －/－ | －/－ | －/－ | －/2＋ | ND/ND | －/3＋ | ND/ND |
|  | 24 | －/－ | －/－ | －/－ | －/－ | －/－ | －/3＋ | ND/ND | －/3＋ | ND/ND |
|  | 25 | －/－ | －/－ | －/－ | －/－ | －/－ | －/2＋ | ND/ND | －/4＋ | ND/ND |
| Control (group 6) | 26 | －/－ | －/－ | －/－ | －/－ | －/－ | －/－ | ND/ND | －/－ | ND/ND |
|  | 27 | －/－ | －/－ | －/－ | －/－ | －/－ | －/－ | ND/ND | －/－ | ND/ND |
|  | 28 | －/－ | －/－ | －/－ | －/－ | －/－ | －/－ | ND/ND | －/－ | ND/ND |
|  | 29 | －/－ | －/－ | －/－ | －/－ | －/－ | －/－ | ND/ND | －/－ | ND/ND |
|  | 30 | －/－ | －/－ | －/－ | －/－ | －/－ | －/－ | ND/ND | －/－ | ND/ND |

Note: ND: no done; ＋: positive; －: negative. HP-PRRSV/PCV2. There are four signal intension levels of detection in the table, “4＋” denotes “100% ≥ intension > 75%”; by parity of reasoning, “3＋” is “75% ≥ intension > 50%”; “2＋” is “50% ≥ intension > 25%”; and “＋” is “25% ≥ intension > 0”. The intension value is the ratio of sample signal value to the positive control signal value.
