# Supplementary material for: Synergistic effects of sequential infection with highly pathogenic porcine reproductive and respiratory syndrome virus and porcine circovirus type 2
Source: Virol J. 2013 Aug 26;10:265. doi: 10.1186/1743-422X-10-265 (PMC3847690; doi:10.1186/1743-422X-10-265)
Supplement: Additional file 3: Table S3 — Detection of HP-PRRSV and PCV2 in each organ of each infected group by RT–PCR/PCR. [file 1743-422X-10-265-S3.doc]

**Additional file 3 –Table S3.**

Detection of HP-PRRSV and PCV2 in each organ of each infected group by RT–PCR/PCR.

| Group | No. | Organ | | | | | | | | | |
| --- | --- | --- | --- | --- | --- | --- | --- | --- | --- | --- | --- |
|  |  | Heart | Liver | Spleen | Lung | Kidney | Tonsil | Inguinal lymph node | Mandibular lymph node | Duodenum | Cerebrum |
| HP-PRRSV/PCV2  (group 1) | 12 | 2＋/＋ | ＋/＋ | 4＋/3＋ | 4＋/2＋ | 2＋/2＋ | 4＋/3＋ | ＋/4＋ | 3＋/2＋ | ＋/2＋ | ＋/＋ |
|  | 13 | ＋/＋ | 2＋/＋ | 3＋/3＋ | 4＋/＋ | ＋/3＋ | 3＋/4＋ | ＋/4＋ | 3＋/3＋ | ＋/＋ | ＋/＋ |
|  | 16 | 2＋/2＋ | ＋/2＋ | 3＋/3＋ | 4＋/＋ | 2＋/3＋ | 4＋/3＋ | 2＋/3＋ | 4＋/2＋ | ＋/＋ | ＋/＋ |
|  | 17 | 2＋/2＋ | 2＋/＋ | 4＋/4＋ | 4＋/2＋ | ＋/3＋ | 3＋/4＋ | 3＋/4＋ | 3＋/3＋ | ＋/＋ | ＋/＋ |
|  | 18 | ＋/＋ | ＋/2＋ | 4＋/3＋ | 4＋/2＋ | ＋/4＋ | 4＋/3＋ | 2＋/4＋ | 4＋/3＋ | ＋/2＋ | ＋/＋ |
| PCV2/HP-PRRSPV  (group 2) | 1 | ＋/＋ | ＋/＋ | 3＋/2＋ | 3＋/＋ | ＋/2＋ | 3＋/2＋ | 2＋/4＋ | 3＋/3＋ | ＋/2＋ | ＋/＋ |
|  | 2 | ＋/2＋ | 2＋/＋ | 4＋/3＋ | 4＋/＋ | ＋/2＋ | 2＋/2＋ | 2＋/3＋ | 3＋/2＋ | ＋/＋ | ＋/＋ |
|  | 3 | ＋/＋ | ＋/2＋ | 3＋/3＋ | 3＋/＋ | ＋/3＋ | 3＋/3＋ | ＋/4＋ | 2＋/2＋ | ＋/＋ | ＋/＋ |
|  | 4 | 2＋/＋ | ＋/＋ | 3＋/2＋ | 4＋/＋ | ＋/2＋ | 4＋/2＋ | 2＋/3＋ | 3＋/＋ | ＋/＋ | ＋/＋ |
|  | 5 | 2＋/＋ | ＋/＋ | 2＋/3＋ | 3＋/＋ | 2＋/2＋ | 3＋/3＋ | ＋/3＋ | 3＋/2＋ | ＋/2＋ | ＋/＋ |
| HP-PRRSV+PCV2  (group 3) | 6 | 2＋/＋ | ＋/2＋ | 4＋/3＋ | 4＋/＋ | ＋/3＋ | 3＋/3＋ | 2＋/2＋ | 2＋/3＋ | ＋/＋ | ＋/＋ |
|  | 8 | ＋/＋ | ＋/＋ | 4＋/3＋ | 3＋/＋ | ＋/2＋ | 3＋/2＋ | 2＋/4＋ | 3＋/2＋ | ＋/＋ | ＋/＋ |
|  | 9 | 2＋/2＋ | 2＋/＋ | 3＋/2＋ | 3＋/＋ | 2＋/＋ | ＋/2＋ | 3＋/3＋ | 3＋/＋ | ＋/＋ | ＋/＋ |
|  | 10 | ＋/＋ | ＋/＋ | 2＋/3＋ | 4＋/＋ | ＋/3＋ | 2＋/3＋ | 2＋/3＋ | 2＋/＋ | ＋/2＋ | ＋/＋ |
|  | 11 | ＋/＋ | ＋/＋ | 2＋/2＋ | 3＋/＋ | ＋/＋ | 4＋/2＋ | 2＋/4＋ | 3＋/3＋ | ＋/＋ | ＋/＋ |
| HP-PRRSV  (group 4) | 7 | ＋/－ | ＋/－ | 3＋/－ | 3＋/－ | ＋/－ | 3＋/－ | 2＋/－ | 2＋/－ | ＋/－ | ＋/－ |
|  | 14 | ＋/－ | ＋/－ | 3＋/－ | 4＋/－ | ＋/－ | 2＋/－ | ＋/－ | 2＋/－ | ＋/－ | ＋/－ |
|  | 15 | ＋/－ | ＋/－ | 2＋/－ | 3＋/－ | ＋/－ | 3＋/－ | 2＋/－ | 3＋/－ | ＋/－ | ＋/－ |
|  | 19 | ＋/－ | ＋/－ | 2＋/－ | 3＋/－ | ＋/－ | 2＋/－ | ＋/－ | 2＋/－ | ＋/－ | ＋/－ |
|  | 20 | ＋/－ | ＋/－ | 3＋/－ | 3＋/－ | ＋/－ | 3＋/－ | ＋/－ | 2＋/－ | ＋/－ | ＋/－ |
| PCV2  (group 5) | 21 | －/＋ | －/＋ | －/2＋ | －/＋ | －/＋ | －/2＋ | －/2＋ | －/＋ | －/＋ | －/＋ |
|  | 22 | －/＋ | －/＋ | －/2＋ | －/＋ | －/2＋ | －/2＋ | －/3＋ | －/2＋ | －/＋ | －/＋ |
|  | 23 | －/＋ | －/＋ | －/2＋ | －/＋ | －/2＋ | －/＋ | －/2＋ | －/＋ | －/＋ | －/＋ |
|  | 24 | －/＋ | －/＋ | －/3＋ | －/＋ | －/2＋ | －/2＋ | －/3＋ | －/＋ | －/＋ | －/＋ |
|  | 25 | －/＋ | －/＋ | －/2＋ | －/＋ | －/2＋ | －/2＋ | －/2＋ | －/2＋ | －/＋ | －/＋ |
| Control  (group 6) | 26 | －/－ | －/－ | －/－ | －/－ | －/－ | －/－ | －/－ | －/－ | －/－ | －/－ |
|  | 27 | －/－ | －/－ | －/－ | －/－ | －/－ | －/－ | －/－ | －/－ | －/－ | －/－ |
|  | 28 | －/－ | －/－ | －/－ | －/－ | －/－ | －/－ | －/－ | －/－ | －/－ | －/－ |
|  | 29 | －/－ | －/－ | －/－ | －/－ | －/－ | －/－ | －/－ | －/－ | －/－ | －/－ |
|  | 30 | －/－ | －/－ | －/－ | －/－ | －/－ | －/－ | －/－ | －/－ | －/－ | －/－ |

Note: ＋: positive; －: negative. HP-PRRSV/PCV2. There are four signal intension levels of detection in the table: “4＋” denotes “100% ≥ intension > 75%”; by parity of reasoning, “3＋” is “75% ≥ intension > 50%”; “2＋” is “50% ≥ intension > 25%”; and “＋” is “25% ≥ intension > 0”. The intension value is the ratio of the sample signal value to the positive control signal value.
